# Application of Balanced Mix Design Methodology to Optimize Surface Mixes with High-RAP Content

**DOI:** 10.3390/ma13245638

**Published:** 2020-12-10

**Authors:** Fabrizio Meroni, Gerardo W. Flintsch, Brian K. Diefenderfer, Stacey D. Diefenderfer

**Affiliations:** 1 Center for Sustainable Transportation Infrastructure, Virginia Tech Transportation Institute, 3500 Transportation Research Plaza, Blacksburg, VA 24061, USA; gflintsch@vtti.vt.edu; 2 Virginia Transportation Research Council, 530 Edgemont Rd, Charlottesville, VA 22903, USA; brian.diefenderfer@vdot.virginia.gov (B.K.D.); stacey.diefenderfer@vdot.virginia.gov (S.D.D.)

**Keywords:** pavement recycling, RAP, surface mix, balanced mix design, laboratory performance

## Abstract

The most common use of reclaimed asphalt pavement (RAP) is in the lower layers of a pavement structure, where it has been proven as a valid substitute for virgin materials. The use of RAP in surface mixes is more limited, since a major concern is that the high-RAP mixes may not perform as well as traditional mixes. To reduce risks or compromised performance, the use of RAP has commonly been controlled by specifications that limit the allowed amount of recycled material in the mixes. However, the ability to include greater quantities of RAP in the surface mix while maintaining a satisfying field performance would result in potential cost savings for the agencies and environmental savings for the public. The main purpose of this research was to produce highly recycled surface mixes capable of performing well in the field, verify the performance-based design procedure, and analyze the results. To produce the mixes, a balanced mix design (BMD) methodology was used and a comparison with traditional mixes, prepared in accordance with the requirements of the Virginia Department of Transportation’s volumetric mix design, was performed. Through the BMD procedure, which featured the indirect tensile cracking test for evaluating cracking resistance and the Asphalt Pavement Analyzer (APA) for evaluating rutting resistance, it was possible to obtain a highly recycled mix (45% RAP) capable of achieving a better overall laboratory performance than traditional mixes designed using volumetric constraints while resulting in a reduction in production cost.

## 1. Introduction

When the Superpave mix design system was introduced in 1993, it featured performance tests that supplemented a series of material specifications and volumetric requirements [1]. However, due to the high cost and complexity that the testing required, the majority of state agencies adopted only the volumetric part of the design process. Over the years, as Superpave mixes were produced and constructed, each state adjusted the volumetric requirements to improve field performance. One of the most common concerns relates to the mixes’ low asphalt content. The general concern was that the Superpave design resulted in dry mixes that were susceptible to cracking while showing a satisfying performance in relation to rutting [2]; to counteract this trend, transportation agencies have modified mix design parameters such as the number of gyrations to allow higher asphalt contents [2]. To supplement the design process, simple performance tests, such as the triaxial dynamic modulus and triaxial static creep tests, were also recommended [3].

In parallel to concerns about low asphalt content, agencies started using larger amounts of recycled materials (i.e., RAP and reclaimed asphalt shingles [RAS]). This has been linked to mix deterioration due to cracking [4,5]. In order to keep the cracking performance under control and allow for the inclusion of recycled materials in the mixes, many cracking tests have been evaluated, such as the Indirect Tensile (IDT) test and Semi-Circular Bending (SCB) test [6,7,8]. Moreover, other non-conventional materials, such as additives, rubber, and rejuvenators, have been increasingly used and have further changed the typical mixes’ behavior. Therefore, alongside cracking tests, evaluation of rutting at the design stage also began, and the idea of a balanced mix design (BMD) took shape [9]. BMD generally features at least two performance tests, one for cracking resistance and the other for rutting, to determine how a mix resists various distresses [10].

With consistently stagnant or shrinking budgets, agencies are aiming to include growing quantities of recycled and non-conventional materials in their mixes. However, the use of such mixes leaves open questions about the impact on long-term performances. To reduce those risks, agencies use specifications that limit the maximum percentages allowed. One of the tools that would support the extensive use of these mixes is BMD. Compared to the volumetric-only mix design, BMD can obtain an indication of mixture behavior by testing in the laboratory at the design stage. This is expected to provide more confidence about the expected mix performance in the field. For instance, the New Jersey Department of Transportation (NJDOT) established a mix design procedure which features at least one of the following three laboratory performance tests: Asphalt Pavement Analyzer (APA) to test rutting resistance, fatigue to evaluate fatigue life, and Overlay Tester (OT) to test cracking resistance [11]; the contractors must submit their loose mixes to the NJDOT Bureau of Materials, which checks if the test specimens meet the required performance criteria. Meanwhile, the Texas DOT uses a BMD procedure with the Hamburg Wheel-Track Test to evaluate the rutting resistance, and OT to test cracking resistance [12].

Currently, in Virginia, the maximum percentages by weight of the mixture of RAP allowed in asphalt mixtures are 30% for surface and intermediate mixes, 35% for base mixes, and 20% for stone matrix asphalt [13]. As a means to address interest in increased RAP percentages, the Virginia Department of Transportation (VDOT) aims to use three performance tests to evaluate performance at the mix design stage: indirect tensile cracking test for cracking resistance, APA for rutting resistance, and Cantabro for durability. These tests were selected based on their practicality (short specimens’ preparation time and high testing speed) and the cost of the necessary equipment [14].

## 2. Purpose and Scope

The main purpose of this research was to produce high recycled content surface mixes capable of performing well in the field, verify the performance-based design procedure, and analyze the results. To produce the mixes, a BMD methodology was used and a comparison with traditional mixes was performed. In particular, two mixes, which acted as control mixes, were prepared in accordance with the requirements of the VDOT volumetric mix design, which originated from the Superpave system. Two additional mixes were designed following a BMD procedure, which featured the indirect tensile cracking test for evaluating the cracking resistance [15] and the Asphalt Pavement Analyzer (APA) for evaluating the rutting resistance [16].

Additionally, the goal was to explore the feasibility of exceeding the limitations of the volumetric mix design, moving towards a performance-based mix design. From this perspective, the results of the laboratory tests would become the main indicators of how to increase the RAP content while obtaining satisfying performances.

## 3. Significance of the Study

Today, the most established use of RAP is in the lower layers of a pavement structure, where it has been proven as a valid substitute for virgin materials. For instance, base layers can achieve excellent performances while including up to 100% RAP through a cold process in which virgin asphalt is added as a recycling agent [17,18]. On the contrary, the use of RAP in hot asphalt surface mixes is more limited, with a major concern being that high RAP mixes may not perform as well as traditional mixes [19]. To reduce risks of compromised performance, the use of RAP has commonly been controlled by specifications that limit the allowed amount of recycled material in the mixes [6].

Compared to the bottom layers, the surface mix has to better withstand higher stresses and aging. Current specifications typically require that the mix components of surface layers (both aggregates and virgin binder) need to be composed of higher percentages of virgin materials, as they are thought to be of higher quality. Being able to include greater quantities of RAP in the surface mix while maintaining a satisfying field performance would support the achievement of potential cost savings for the agencies [20] and environmental savings for the public. A performance-based mix design procedure, such as BMD, is one of the most promising tools that agencies can use to expand the use of RAP and reduce the risks of using high recycled content mixtures. In fact, BMD allows control of the recycled mixture resistance to distresses and compares it to mixes having lower recycled contents at the design stage. It must be considered that, in light of an initial low cost, it is the mixture’s ability to resist distresses over its life cycle that eventually determines the overall costs of a recycled pavement and its potential to achieve economic savings over its entire lifespan.

## 4. Methodology

First, the research team defined a high-RAP-content control mix which met the current VDOT specifications. The mix, which will be referred to as 30-Superpave, contained 30% RAP by weight of total mixture, which is the current upper limit for surface mixes [13]. An additional control mix, referred to as 45-Superpave, was designed to match the aggregate structure and binder content of 30-Superpave but at an RAP content of 45% by weight of total mixture.

Based on the performance information (obtained through the indirect tensile test and APA rut test) of the afore-mentioned mixes, changes to the mix composition were made. Two more mixes were designed with a different aggregate structure, which did not meet the VDOT specification requirements but met the current tentative BMD thresholds [21]. The new mixes contained 30% and 45% RAP by weight of total mixture and were designated as 30-BMD and 45-BMD, respectively. The optimum binder content of the two mixes was determined based on balancing cracking and rutting resistance. The changes in the mix composition and binder content were determined with the goal of improving the mixes’ performance, even if the final properties of the mix did not satisfy the existing volumetric requirements.

## 5. Review of High RAP Mix Design

The inclusion of RAP in hot mix asphalt designed using the Superpave procedure was formalized through NCHRP Project 9–12, in which a three-tier system was developed. The system was based on the properties of the hardened RAP binder and blending charts were developed for high RAP contents [22,23]. Through the following NCHRP Project 9–46, which was aimed at improving the recycling practice, the design of high RAP mixes was guided by the RAP binder ratio [23]. In both cases, recovery and grading of the aged binder were key steps in the design process.

To include higher RAP quantities, recycling agents such as rejuvenators and softening agents have also been introduced. Rejuvenators are chemical agents capable of restoring the physical and chemical properties of the old binder. They differ from softening agents, which lower the viscosity of the aged binder [24]. It is important to select the right rejuvenating agent, based on its compatibility with the aged binder [25]. A lot of uncertainties still remain over the properties of the rejuvenated RAP binder; therefore, RAP has often been used in lower-level applications without fully exploiting the value of the asphalt binder available in the RAP [26].

Over time, it has been possible to observe that there is no final agreement between researchers with respect to the impact of RAP on field performances:

- Huang, et al. [27] conducted a laboratory study and reported that up to 20% RAP improved the tensile strength and fracture resistance, while 30% RAP changed the performance significantly;
- Kim, Byron, Sholar and Kim [5] reported that mixes with more RAP showed higher rutting resistance and lower fracture energy. However, for high-RAP mixes, the use of softer binders could reduce the rutting performance;
- Widyatmoko [28] indicated that mixtures containing RAP tend to have lower stiffness, lower resistance to permanent deformation, and better resistance to fatigue than equivalent mixtures without RAP. This behavior would be explained by the use of softer binders and rejuvenating agents;
- Apeagyei, et al. [29] reported similar rutting performances at the lower (0%) and higher (25%) RAP contents, while the best performances corresponded to mixtures that contained intermediate amounts of RAP (10% and 15%);
- Al-Qadi, Aurangzeb, Carpenter, Pine and Trepanier [4] reported that, as RAP content increased, complex modulus, tensile strength, moisture damage resistance, fatigue life, and rutting resistance increased. On the contrary, the thermal cracking resistance decreased;
- McDaniel, et al. [30] found that RAP increased the stiffness of the mixes while improving the fatigue life and not affecting the thermal cracking resistance;
- Izaks, et al. [31] observed how high RAP content mixtures had a higher resistance to rutting when compared to reference traditional mixes. The recycled mixes exhibited similar mechanical properties, such as resistance to fatigue and stiffness.

Many factors contribute to the properties of an asphalt mix and, by consequence, do not allow for generalized conclusions on the RAP effects: the inherent variability of RAP, the difficulty of defining the interaction level of the virgin binder, recycled binder and additives, and the open questions concerning rejuvenators have led to the definition of new mix design methods [10]. The BMD concept was introduced to design mixes as the best compromise between rutting resistance and cracking resistance [9]. While increasing the confidence in the final properties of the mix, BMD is not necessarily linked with the evaluation of the RAP binder grade and the interaction level between the recycled binder and virgin binder.

Laboratory performance tests must be associated with appropriate management of the RAP. It is fundamental that the material used during the design stage maintains consistent properties throughout the production process. To support the construction of highly recycled mixes, RAP needs to be appropriately processed, crushed, and screened. RAP stockpiles need to be regularly sampled for quality control to ensure that consistent gradation and binder content are maintained [32].

## 6. Mixes Properties

**Figure 1:**
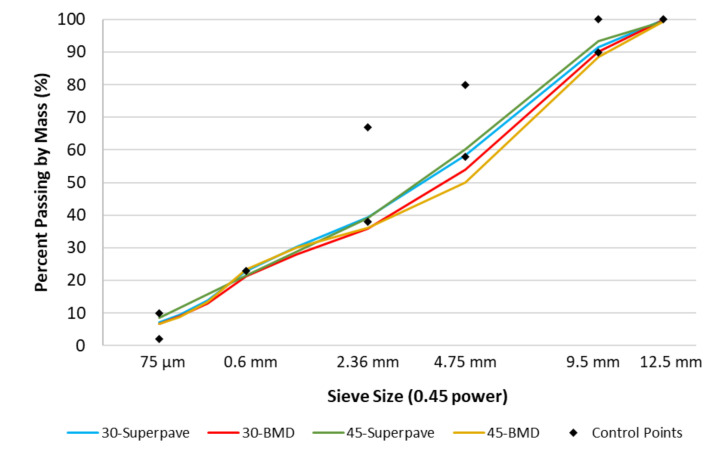
Gradation curves.

All the evaluated mixes had a nominal maximum aggregate size (NMAS) equal to 9.5 mm, and the selected binder performance grade (PG) was 64-22. The volumetric parameters of the control mixes are reported in Table 1, while the gradation curves of the mixes are reported in Figure 1. It is possible to observe how both control mixes were designed in accordance with VDOT’s volumetric requirements. The 30-BMD and 45-BMD mixes were instead designed with the goal of obtaining a coarser mix allowing less passing at the sieve sizes of 4.75 and 2.36 mm.

## 7. Laboratory Aging of the Specimens

**Figure 2:**
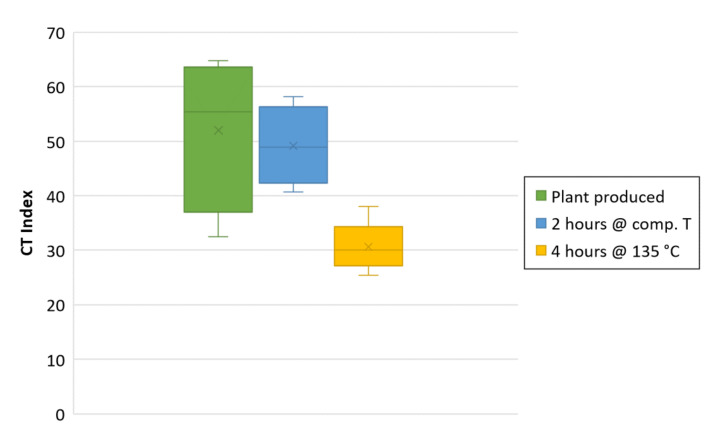
Aging impact on recorded cracking resistance of the control mix (30-Superpave). The middle line of the box represents the median, the x in the box represents the mean (four specimens per aging type).

To analyze mix performance at the mix design stage, appropriate aging needs to be applied to the specimens produced in the laboratory. The American Association of State Highway Transportation Officials (AASHTO) recommends 4 h of aging at 135 °C [33]. VDOT modified this by requiring 4 h of aging at the mix design compaction temperature. However, there is still considerable discussion in the community regarding the most appropriate aging protocol to simulate plant-produced material. To determine the most appropriate way of aging the samples during the study, plant-produced samples of the control mix were collected as a reference to compare the cracking resistance results due to the different aging techniques. Both 2 and 4 h of aging were evaluated. The results are shown in Figure 2.

Based on the comparison with specimens taken directly from the plant, the 2-h aging produced more similar results, while the 4-h aging significantly reduced the laboratory performances of the mixes. For this reason, the aging applied to specimens during the study was 2 h at compaction temperature.

## 8. Performance Optimization Process

**Figure 3:**
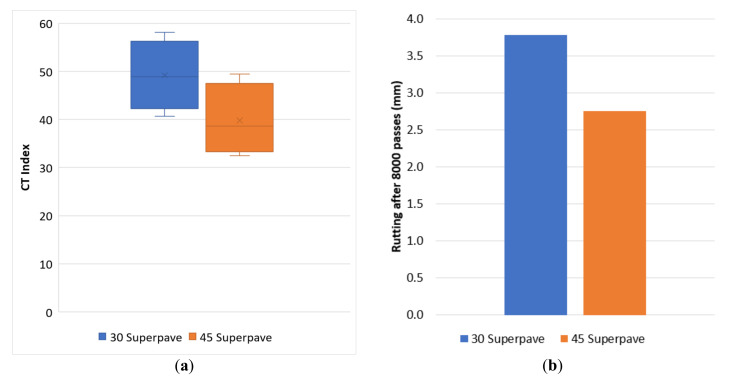
CT Index (**a**) and APA (**b**) results for control mixes.

The performance evaluation was first conducted on the control mixes, with respect to both cracking and rutting resistance, evaluated through the indirect tensile test and APA, respectively. In accordance with the test methods [15,16], all specimens were compacted at 7% air void content with a 0.5% tolerance. The results are shown in Figure 3. While there are still no common guidelines on the definition of performance requirements, the VDOT is looking to establish thresholds to identify satisfactory levels of performance in the field. In particular, for the CT Index, the proposed minimum value is 70, while the measured rut depth after 8000 passes in the APA should be less than 8 mm [21].

Both mixes met the volumetric specifications; however, Figure 3 shows that the cracking resistance minimum was not met while the rutting resistance test result was well below the proposed maximum limit. This behavior has been associated with Superpave mixes [34]. Even with a limited impact, the inclusion of 45% RAP in the mix corresponded to lower cracking resistance and higher rutting resistance. These trends have been traditionally associated with high RAP contents and have also been confirmed by various studies presented in the literature [4,5].

**Figure 4:**
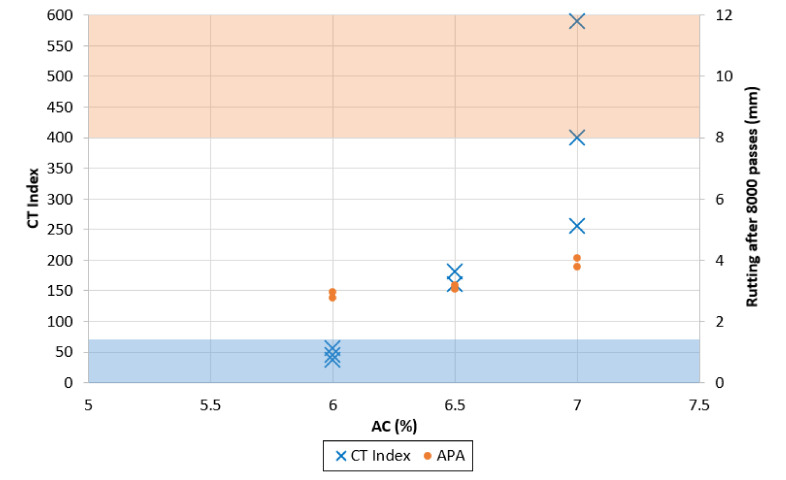
Performance tests on mix 30-BMD.

**Figure 5:**
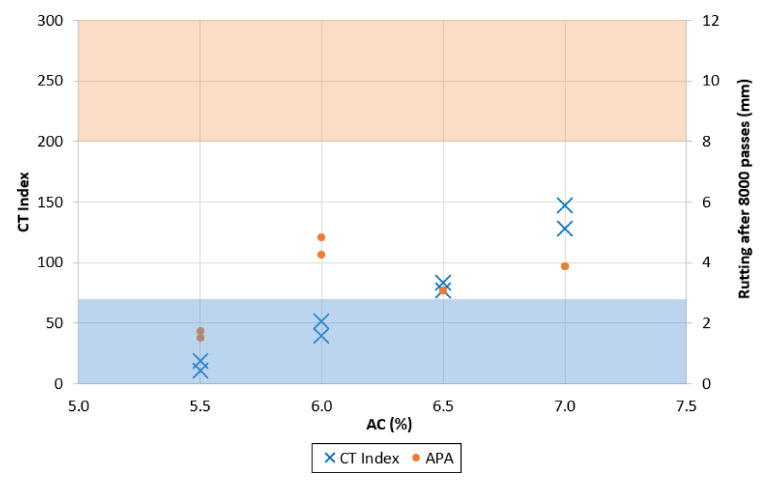
Performance tests on mix 45-BMD.

To improve the mixes’ cracking resistance and obtain better overall performance while increasing the RAP content, a different aggregate structure was studied and tested at different asphalt contents. Figure 4 and Figure 5 show the performance test results for the mixes containing 30% and 45% RAP, respectively, which do not meet VDOT gradation requirements. The shaded areas represent the zones in which the mix fails to meet the performance criteria: the orange area highlights rutting depths greater than 8 mm, while the blue area shows CT Index values lower than 70.

The definition of optimum binder content was based on balancing both performance requirements. As expected, the impact of increasing the asphalt content was noticeable with regard to both cracking and rutting. As the mixture binder content increased, the mix became less stiff, allowing it to have higher cracking resistance. The mix containing 30% RAP showed the potential of reaching very high values of cracking resistance, while the 45% RAP mix appeared to be more limited. This limitation can be explained by the partial contribution of the RAP binder: when comparing the two mixes with the same binder content design value, the partial contribution of the RAP binder is going to appear, with a greater impact on the 45% RAP mix. The 45-BMD mix showed an increase in cracking resistance with respect to increasing binder content, while the rutting susceptibility is not consistent with increasing binder content. For 30-BMD, higher binder contents corresponded to a higher cracking resistance, with a measured maximum CT Index value at 7% AC more than ten times larger the maximum measured at 6% AC. The rutting resistance for 30-BMD showed a slight deteriorating trend, however, the rutting resistance was well below the 8-mm limit in all cases.

The test results indicated that to achieve satisfying performances, the minimum design AC for 30-BMD was approximately 6.2%, while for 45-BMD it was 6.5%. The design asphalt contents were selected based on compliance with the minimum CT Index criteria plus a safety factor. Both cases represented a meaningful improvement in terms of performance when compared to the control mixes.

**Figure 6:**
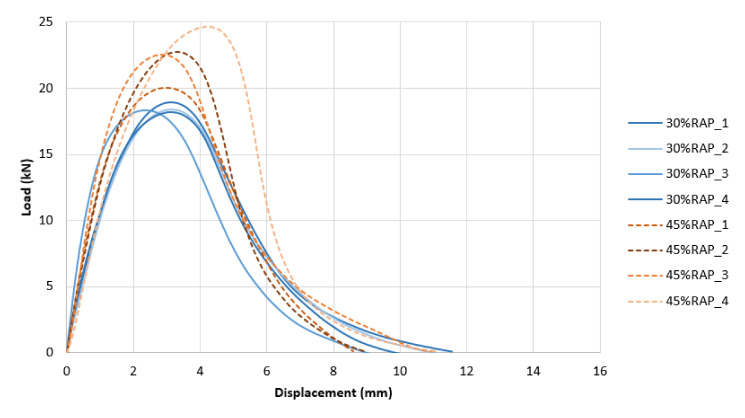
Load vs. Displacement Curves: 30-Superpave, 45-Superpave.

**Figure 7:**
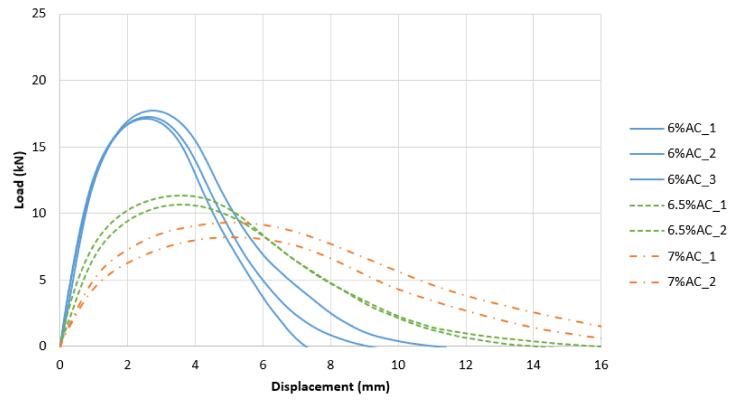
Load vs. Displacement Curves: 30-BMD.

**Figure 8:**
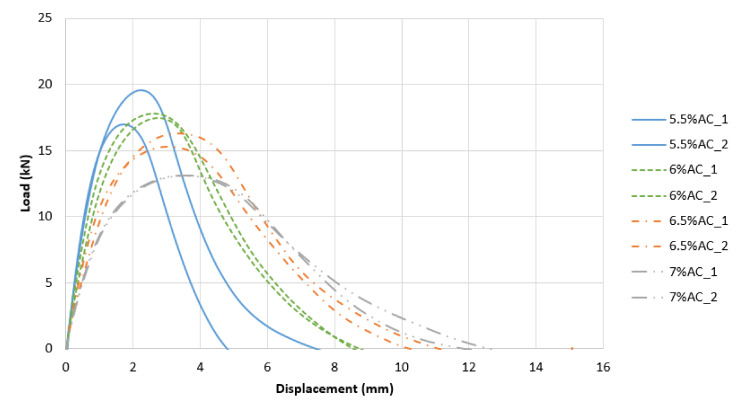
Load vs. Displacement Curves: 45-BMD.

To better visualize the different performances with respect to cracking resistance, Figure 6, Figure 7 and Figure 8 show the various load vs. displacement curves. It is possible to observe how the Superpave mixes exhibited a generally higher peak load and a more brittle behavior than the optimized mixes. The BMD mixes had a more ductile behavior, especially as the AC increased.

## 9. Cost Comparison

To further examine the feasibility of implementing the BMD mix design process rather than the volumetric requirements, a cost analysis was conducted to evaluate the impact on the mixes’ production cost. For both mixes, 30-BMD and 45-BMD, the design AC was selected so that it was possible for the BMD mixes to outperform the control mixes and achieve acceptable levels of cracking and rutting resistance with respect to the VDOT-proposed requirements. A summary of the mixes’ composition is shown in Table 2.

The material costs and final estimates are summarized in Table 3. To calculate the costs of the various mix components, representative Virginia statewide averages were used and assumptions on the mixture constituents’ costs were used. In particular, the aggregate costs were discounted by 50% to account for their use in asphalt mixture production. For the RAP, while the purchase cost is considered equal to zero, processing-related costs were considered. For this analysis, the quantity of the required virgin binder was calculated as the design AC minus the quantity of binder provided by the RAP.

**Figure 9:**
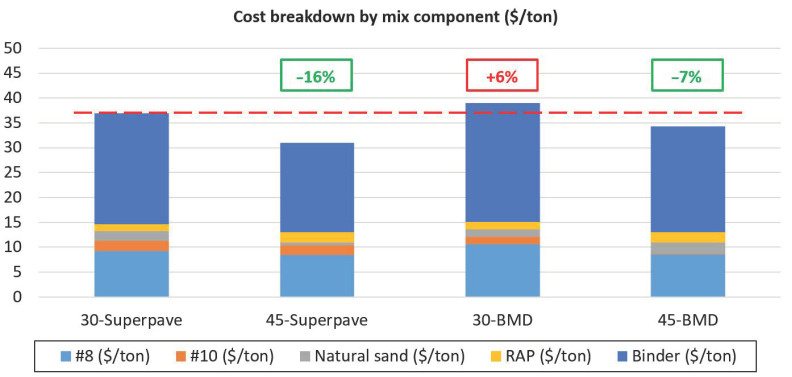
Mix cost comparison.

In general, as shown by the comparison between the two control mixes in Figure 9, the increased use of RAP resulted in a reduction in virgin aggregates and binder. However, to pass the requirements of the laboratory optimization process, the mix 30-BMD required more virgin binder content than the control mix, resulting in a higher production cost (+6%) when compared to the control mixture with the same RAP content. With respect to the 45% RAP-optimized mix, it was possible to achieve significant savings (7%) compared to the 30-Superpave mix, even if the design binder content of the BMD mix was higher.

To further examine the economic feasibility of the BMD process, a simplified life-cycle cost analysis (LCCA) was conducted. The LCCA examined the impact of production and operating (maintenance) costs over a period of 30 years. The control mix 30-Superpave was used as a reference, with a service life varying between 8 and 12 years. At the end of the service life, mill and overlay operations were planned for all mixes. The scheduled operations over the course of the 30-year analysis period were discounted at a rate of 4%. Different scenarios were evaluated for both the optimized mixes, which supposedly would achieve extended service lives, thus requiring fewer maintenance operations. Four different scenarios of service life variation were evaluated, in which the cost change of each optimized mix was analyzed from a decrease of 1 year to an increase of 2 years. The results are shown in Table 4 (30-BMD) and Table 5 (45-BMD).

It is possible to observe how, for 30-BMD, a life extension of 1 year is enough to present a more convenient solution, with the exception of the scenario in which the reference mix, 30-Superpave, provides a service life equal to or greater than 12-years. Instead, 45-BMD can achieve cost savings even if the service life is 1 year shorter than the control mix (when compared to 30-Superpave’s service lives, which are equal to or greater than 11-years).

## 10. Conclusions

In this study, it was possible to investigate how the implementation of a BMD system could represent a significant upgrade of the current design practice. Through this performance-based procedure, it was possible to obtain a high recycled content mix, such as 45-BMD, which would provide a better overall performance while providing a reduction in production cost when compared to traditional mixes. In addition to the economic savings, the higher RAP content improves the environmental impact of the mix as it uses less virgin materials. Overall, the following conclusions were drawn:

- The use of gradation and volumetric requirements did not guarantee a satisfactory performance (in terms of laboratory cracking and rutting resistance) for the 30-Superpave and 45-Superpave mixes, which were designed in accordance with Superpave requirements;
- The mixtures designed using the Superpave gradation and volumetric requirements were outperformed in the laboratory by the selected BMD mixes;
- As expected, for both the control and optimized mixes, the inclusion of higher RAP contents corresponded to lower cracking resistance and higher rutting resistance in the laboratory;
- Even if high RAP contents may require higher asphalt contents to achieve satisfactory cracking resistance, the impact on rutting performance was very limited for the mixtures evaluated in this study;
- Compared to the control mixes, the optimized mixes showed potential economic savings. The 45-BMD mix resulted in lower production costs and presented a better laboratory performance than the control mixes. A simplified LCCA showed how a 1-year service life extension would be enough to justify the higher production cost of 30-BMD. The LCCA also showed that, when compared to the control mix 30-Superpave, 45-BMD would allow for achieving significant savings, even if it would not extend the service life.

## 11. Recommendations

Based on the findings of this research, the following recommendations are made:

- The study used two simple performance tests to measure the cracking and rutting resistance of the mixes. However, the selection of appropriate laboratory tests is fundamental for the effectiveness of a BMD approach. In particular, field performance is needed to verify the conclusions that are based on laboratory testing. Techniques like pavement recycling are generally promoted because of their economic savings and the environmental benefits they entail. However, only if the field performances are adequate would it be possible to fully take advantage of the recycling process. If the RAP inclusion results in a shorter pavement lifespans or inappropriate performance, the initial benefits would become irrelevant due to the necessity of additional maintenance;
- The RAP binder content affects the mixture’s final asphalt content, which may result in the mix being higher or lower than the designed AC. If the asphalt content of the RAP changes, the mixes could become under-asphalted or over-asphalted, resulting in a poor pavement performance. Therefore, because of its inherent variability, the properties (e.g., gradation and AC) of the RAP taken from the plant stockpiles need to be checked throughout the process. The need to track the sources of the RAP and maintain separate stockpiles should be investigated. Changes in the binder content of the RAP source stockpile and the effects of using multiple RAP stockpiles should be investigated to determine the influence on producing a consistent mixture with high recycled contents;
- Even though part of the aged RAP binder contributes to the mix properties, the 45% RAP mix required a higher overall AC percentage than the 30% RAP to achieve the same level of performance. This is because, at higher RAP contents, the aged binder contributes to a larger proportion of the mixture properties. For this reason, the use of rejuvenators should be investigated to further optimize the necessary quantity of virgin binder, especially at high RAP contents.

## Figures and Tables

**Table 1:** Control mixes’ properties.

	30-Superpave	45-Superpave	Criteria [13]
G_mm_^(a)^	2.492	2.518	-
VFA (%) ^(b)^	77.2	77.2	75–80
VMA (%) ^(c)^	16.5	16.6	16 (min)
Fines/Asphalt Ratio	1.2	1.3	0.7–1.3

^(a)^ Theoretical Maximum Specific Gravity. ^(b)^ Voids Filled with Asphalt. ^(c)^ Voids in the Mineral Aggregate.

**Table 2:** Breakdown of mixes’ composition.

Mix Component	Mix			
	30-Superpave	45-Superpave	30-Optimized	45-Optimized
Aggregate No. 8	43	39	50	40
Aggregate No. 10	14	13	10	0
Natural sand	13	3	10	15
RAP	30	45	30	45
Asphalt Content ^(a)^	5.9	5.9	6.2	6.5

^(a)^ Sum of virgin binder and binder included in the RAP.

**Table 3:** Mixes’ cost comparison.

Mix Component	Cost ($/ton)	Mix			
		30-Superpave	45-Superpave	30-Optimized	45-Optimized
Aggregate No. 8 ^(a)^	22.50	9.23	8.44	10.68	8.60
Aggregate No. 10 ^(b)^	15.00	2.06	1.96	1.49	0.12
Natural sand ^(b)^	15.00	1.92	0.55	1.49	2.23
RAP ^(c)^	5.00	1.41	2.12	1.41	2.10
Binder cost ^(d)^	528.55	22.38	17.98	23.99	21.23
Cost per US ton (USD)		37.00	31.05	39.06	34.29
Cost per lane (USD/mile) ^(e)^		895.38	751.40	945.22	829.86

^(a)^ Adjusted Virginia statewide averages (1 January 2018 through 1 February 2020). ^(b)^ Adjusted Virginia statewide averages (1 November 2016 through 1 December 2018). ^(c)^ The RAP purchase cost is assumed equal to zero, the cost listed is related to the RAP processing phase. ^(d)^ VDOT asphalt price (PG 64S-22, April 2020). The calculated cost is relative only to the virgin binder. ^(e)^ Assumption of 165 lb. of mix per yd^3^, with a layer thickness of 1.5 in.

**Table 4:** Cost increase/reduction in 30-BMD with respect to 30-Superpave (%).

Service Life 30—Superpave (Years)	Service Life Change 30—BMD (Years)			
	−1	+0	+1	+2
8	+16.6%	+5.6%	−3.2%	−10.7%
9	+15.1%	+5.6%	−2.6%	−8.4%
10	+14.5%	+5.6%	−0.7%	−6.2%
11	+12.2%	+5.6%	−0.3%	−5.5%
12	+11.7%	+5.6%	+0.1%	−4.8%

**Table 5:** Cost increase/reduction in 45-BMD with respect to 30-Superpave (%).

Service Life 30—Superpave (years)	Service Life Change 45—BMD (years)			
	−1	+0	+1	+2
8	+2.3%	−7.3%	−15.0%	−21.6%
9	+1.1%	−7.3%	−14.5%	−19.6%
10	+0.5%	−7.3%	−12.8%	−17.6%
11	−1.5%	−7.3%	−12.4%	−17.0%
12	−1.9%	−7.3%	−12.1%	−16.5%
